# Visualization of melanoma tumor with lectin-conjugated rare-earth doped fluoride nanocrystals

**DOI:** 10.3325/cmj.2014.55.186

**Published:** 2014-06

**Authors:** Tetiana Dumych, Maxym Lutsyk, Mateusz Banski, Antonina Yashchenko, Bartlomiej Sojka, Rostyslav Horbay, Alexander Lutsyk, Rostyslav Stoika, Jan Misiewicz, Artur Podhorodecki, Rostyslav Bilyy

**Affiliations:** 1 Institute of Cell Biology, National Academy of Sciences of Ukraine, Lviv, Ukraine; 2 Danylo Halytsky Lviv National Medical University, Lviv, Ukraine; 3 Wroclaw University of Technology, Institute of Physics, Wroclaw, Poland; * Equally contributed as senior authors.

## Abstract

**Aim:**

To develop specific fluorescent markers for melanoma tumor visualization, which would provide high selectivity and reversible binding pattern, by the use of carbohydrate-recognizing proteins, lectins, combined with the physical ability for imaging deep in the living tissues by utilizing red and near infrared fluorescent properties of specific rare-earth doped nanocrystals (NC).

**Methods:**

B10F16 melanoma cells were inoculated to C57BL/6 mice for inducing experimental melanoma tumor. Tumors were removed and analyzed by lectin-histochemistry using LABA, PFA, PNA, HPA, SNA, GNA, and NPL lectins and stained with hematoxylin and eosin. NPL lectin was conjugated to fluorescent NaGdF_4_:Eu^3+^-COOH nanoparticles (5 nm) via zero length cross-linking reaction, and the conjugates were purified from unbound substances and then used for further visualization of histological samples. Fluorescent microscopy was used to visualize NPL-NaGdF_4_:Eu^3+^ with the fluorescent emission at 600-720 nm range.

**Results:**

NPL lectin selectively recognized regions of undifferentiated melanoblasts surrounding neoangiogenic foci inside melanoma tumor, PNA lectin recognized differentiated melanoblasts, and LCA and WGA were bound to tumor stroma regions. NPL-NaGdF_4_:Eu^3+^ conjugated NC were efficiently detecting newly formed regions of melanoma tumor, confirmed by fluorescent microscopy in visible and near infrared mode. These conjugates possessed high photostability and were compatible with convenient xylene-based mounting systems and preserved intensive fluorescent signal at samples storage for at least 6 months.

**Conclusion:**

NPL lectin-NaGdF_4_:Eu^3+^ conjugated NC permitted distinct identification of contours of the melanoma tissue on histological sections using red excitation at 590-610 nm and near infrared emission of 700-720 nm. These data are of potential practical significance for development of glycans-conjugated nanoparticles to be used for in vivo visualization of melanoma tumor.

Melanomas are considered as one of the most aggressive and fastest growing human tumors, and are characterized by high risk of metastasis and death. Since no effective systemic therapy exists to cure melanoma at an advanced stage, surgical removal of thin early-stage melanoma remains the best solution for recovery. Due to its high metastatic potential, a precise visualization of tumor contours during surgical incision or radio therapy is important to ensure and control its complete removal and save unaffected (healthy) tissue (1,2). So far there have been no markers available that would ensure in vivo visualization of the tumor due to its intracellular accumulation of most molecular markers that are used to detect melanomas (3) and low signal-to-noise ratio of the available labels for imaging tissues.

Optically active inorganic nanocrystals (quantum dots, ie, cadmium-selene, cadmium-sulfur) have recently been widely used in research related to bio-medicine as efficient in vivo and *in vitro* optical markers but also as building blocks in bio-sensors. The main reasons for this are their unique physical and chemical properties. As compared to molecular markers (ie, green fluorescent protein, rhodamine), their emission is a few orders of magnitude stronger, more stable in time (weak blinking, no photobleaching), and can be easily tuned in a wide spectral range simply by changing the quantum dot size. However, even if they are much better from the physico-chemical point of view, from the application point of view, at this moment their clinical use is still questionable mainly because of their high risk of toxicity and lack of strong counter-arguments like their optical activity in infrared spectral range or multifunctionality (4). The infrared activity of optical probes is especially important, since the infrared light excitation and/or emission increases the penetration depth of light into the tissue, reducing scattering and autofluorescence, which all together would make them excellent markers for in vivo imaging.

One of the solutions, combining the advantages of both concepts (quantum dots and molecular markers), is to make nontoxic inorganic nanocrystals doped with lanthanide ions. These markers, because of their lack of chemical toxicity, flexibility in tuning the emission, and excitation properties in a wide spectral range ( ~ 400 -1600 nm) (4) can be designed as multifunctional probes being simultaneously a magnetic probe (to be used in MRI imaging), optical nanothermometer, or other functionalities that can be added to this probe due to its high surface area. In addition, these nanocrystals are characterized by extremely narrow emission lines (a few nm) and long emission decay times (up to a few ms) (4). These parameters make them also excellent candidates for many imaging techniques.

Recently, we have obtained sub-10 nm fluoride nanocrystals doped with Eu^3+^ ions (5), which can be characterized by several emission bands, including a band at 720 nm depending on Eu^3+^ concentration (6). Since the body tissues are permeable to infrared radiation outside the water absorbance region, the near infrared (NIR)-based imaging technologies are of great importance for in vivo medical imaging. One of the main candidates to benefit from NIR imaging would be melanoma, being a primary skin tumor with a superficial localization. This would allow precise determination of tumor margins, with an aim to monitor its spread and control removal efficiency. Here we utilized NaGdF_4_:Eu^3+^ nanocrystals, which can serve as a model of rare-earth NC, possessing one important feature, namely the ability to be easily visualized by detecting their red fluorescence (even by naked eye), and tested the possibility of NIR (or far-red) imaging with 700-720 nm fluorescence, which can be detected with available routine detectors.

To provide a specific but still reversible recognition of the melanoma cells, we screened a set of glycans-recognizing proteins – lectins – for their ability to bind to melanoma cells. Since, usually the lectin affinity binding constant with glycan ( ~ 10^−7^M) is two orders of magnitude lower than that of monoclonal antibodies with corresponding antigen ( ~ 10^−9^M), lectin binding to tissue can be disrupted with the use of specific sugar inhibitors (usually non-harmful, sweet compounds acting at hundred nanomolar range) (7). Glycans posses an enormous amount of information and thus can serve as specific molecular markers of specific cells and tissues (8,9); glycan synthesis is usually very complex and thus is often disrupted in tumor cells, usually providing cells with not fully differentiated glyco-phenotype (8,10). Specific glycosylation changes attributable to melanoma tumors have been reported recently (11,12) and we have effectively used lectin-conjugated rare-earth-based NC for visualization of dying cells (13,14).

Since melanoma has a wide spectrum of histological features that mimic epithelial, hematologic, mesenchymal, and neural tumors (15), we hypothesized that some specific altered glycans existed in melanoma tumor and can serve as sensitive markers of malignant cells.

The following lectins were used to screen tumor-related glycans: *Laburnum anagyroides* bark agglutinin (LABA); *Perca fluviatilis* agglutinin (PFA); Peanut agglutinin (PNA); *Helix pomatia* agglutinin (HPA); *Sambucus nigra* agglutinin (SNA); *Galanthus nivalis* agglutinin (GNA); *Narcissus poeticus* lectin (NPL); *Lens culinaris* agglutinin (LCA); wheat germ agglutinin (WGA). Many of these lectins were first discovered (LABA, PFA) (16) or characterized (GNA, NPL) in our laboratory. Two lectins were selected as promising markers of undifferentiated melanoblasts and differentiated melanoblasts and were further conjugated to the surface of NIR-emitting NC. The NC-lectin conjugates were used for histochemical staining and provided an intense non-photobleaching fluorescent signal in both red and NIR spectral part and high selectivity in visualization of melanoma tissue *in vitro*.

## Materials and methods

### Melanoma tumor growth and induction

The C57BL/6 mouse-derived B16F10 melanoma cell line was propagated in Dulbecco's Modified Eagle Medium (DMEM) supplemented with 10% fetal bovine serum (FBS) and penicillin/streptomycin and was re-seeded every 3 to 5 days, so that a confluent layer was not formed. Cells were grown in culture dishes, detached from cell surface with trypsin/EDTA, washed in DMEM w/o serum, and centrifuged. Cell pellet resuspended at 40 million cells/mL and 100 µL of cell suspension (containing in total 4 million of cells) was injected subcutaneously into the back region of C57BL/6 mice (17,18). Twelve mice (4 w/o NC and 8 injected with NC) were used in the experiments. C57BL/6 mice were bred at the Institute of Cell Biology, Lviv, Ukraine from May to December 2013 and kept on a standard diet with drinking water available *ad libitum*. Animal hair was trimmed at the place of injection and tumor growth was monitored with caliper every 2 days. Tumor growth was monitored every two days by measuring its dimensions and calculating the volume. When the tumor dimensions exceeded 15 mm, the mouse was anesthetized and sacrificed. Animal studies were conducted according to guidelines determined by the Law of the Ministry of Healthcare of Ukraine, No. 281 from November 1, 2011 for the care and use of laboratory animals and were approved by Ethics Council of the Institute of Cell Biology.

### Preparation of histological slides

Mice were sacrificed with cervical dislocation under dimethyl ether anesthesia. The tumors were removed, sliced centromedially, fixed overnight in 4% paraformaldehyde in phosphate buffered saline (PBS), and embedded in paraffin wax according to the standard protocol. For general morphology studies, 5 to 7-µm thick sections were stained with hematoxylin and eosin (HE) (19). Paraffin sections of melanoma tumor were deparaffinized and incubated with NPL-NaGdF_4_:Eu^3+^ NC. After this, samples where thoroughly washed and counterstained with 4',6-diamidino-2-phenylindole dihydrochloride (DAPI) to visualize cell nuclei (if needed – dried and clarified) and mounted.

### Lectin-histochemistry

Deparaffinized sections were incubated for 20 min in methanol containing 0.3% H_2_O_2_ to block the activity of endogenous peroxidase, through graded ethanol brought to PBS pH 7.4, rinsed in three portions of PBS (5 min each), and incubated for 45 min with lectin-peroxidase conjugates (5 μg/mL in PBS) or lectin-biotin conjugates for NPL lectin (5 μg/mL in PBS) in a moist chamber at room temperature. In case of lectin-biotin conjugates, slides were washed twice with PBS and incubated with streptavidin-peroxidase (Vector, Burlingame, CA, USA), 1 µg/mL for 30 min at RT and 3 × washed with PBS solution. Lectin binding sites were visualized in PBS, containing 0.05% diaminobenzidine (Sigma, St. Louis, MO, USA) and 0.015% H_2_O_2_. Thereafter, slides were twice washed in distilled water, and after dehydration mounted in balsam. For semiquantitative evaluation of lectin binding, two investigators performed the analysis independently, blinded to lectin type. Binding intensity was evaluated using a semiquantitative scale as follows: +++ very strong, ++ strong, + weak positive, and - negative labeling (not shown) (Table 1).

**Table 1:** Relative staining of different tumor regions with a panel of used lectins*

Lectin	Undifferentiated melanoblasts around blood vessels	Differentiated melanoblast	Melanocytes	Tumor stroma	Vessel endotelium	Erythrocytes
HPA			++	+		
LCA				+++		
GNA	+	+	++			
LABA			++	+		
WGA	+	+	+	+++	++	
PFA				++	+++	
PNA		++	++			
NPL	+++					+++

*Abbreviations: LABA – *Laburnum anagyroides* bark agglutinin; PFA – *Perca fluviatilis* agglutinin; PNA – Peanut agglutinin; HPA *– Helix pomatia* agglutinin; SNA – *Sambucus nigra* agglutinin; GNA *– Galanthus nivalis* agglutinin; NPL *– Narcissus poeticus* lectin; LCA *– Lens culinaris* agglutinin; WGA – wheat germ agglutinin.

### Conjugation of lectin with NC

The hydrophobic NaGdF_4_:Eu^3+^ nanocrystals were obtained by modified co-thermolysis method described elsewhere (5). Their functionalization to hydrophilic NaGdF_4_:Eu^3+^-COOH form was obtained according to our variant of ligand exchange method, which is patent pending. We added to NC solution in toluene another solution containing hydrophilic ligand and mixed them for some time under inert conditions. In particular, we substituted trioctylphosphine oxide (TOPO) with meso-2,3-dimercaptosuccinic acid (DMSA), which resulted in water dispersible NCs with COOH groups on their surface.

NaGdF_4_:Eu^3+^ - COOH were sonicated and filtered through 0.22 µm-pore filter and transferred to MES 0.1M pH 4.5 buffer. Lectin to be used for conjugation was dissolved in MES, 0.1M, pH 4.5 at 10 mg/mL. 2 mg of protein was added to every 1 mL of NC suspension (containing 10 mg of NC). EDC (1-ethyl-3-(3-dimethylaminopropyl)carbodiimide hydrochloride) was added to the reaction medium to obtain final 2% concentration. Suspension was reacted for 2 h at RT under constant mixing. The obtained suspension was dialyzed against PBS 3 times. For storage purposes, BSA (1% final concentration) and sodium azide (0.05% final concentration) were added to NC suspension. Purification of NPL- NaGdF_4_:Eu^3+^ was conducted in 1% agarose gel with loading of either 20 µL of lectin-NC suspension or 20 µg of lectin per well. Agarose gel electrophoresis was performed according to routine laboratory procedures. Agarose was stained with Coomasie G-250 (Carl Roth, Karlsruhe, Germany) and scanned.

### Fluorescent microscopy

Fluorescent microscopy was performed with fixed slides under a Carl Zeiss AxioImager A1 DIC/fluorescent microscope (Oberkochen, Germany) using 0.4NA 10 × , 0.75 NA 40 × air, and 1.3 NA 100 × oil immersion objectives. Fluorescent images were taken by a Zeiss AxioCam MRmIII cooled digital CCD camera under constant exposure. Fluorescence of NC was evaluated at red channel (610 nm emission was obtained at 532 nm excitation) and at similar conditions the emission at NIR channel (700 nm) was detected. In parallel, the nearest tumor slices were stained with HE and imaged for comparison. HE images were made at the same microscope set up using additional Canon (Tokyo, Japan) CCD RGB camera with constant focus and exposure settings; Zeiss AxioVision and ImageJ software (National Institutes of Health, Bethesda, MD, USA) were used for image analysis. TEM microscopy and spectroscopy analysis were performed as described previously (6). Statistical analysis was performed in Microsoft Excel, using *t* test and *P* < 0.05 was considered significant.

## Results

### Growth of the melanoma tumor

On average, melanoma tumors reached 15 mm in diameter within 17 days (range, 14-24 days). Mean weight (±standard error of the mean) of the tumor was 860 ± 160 mg, and it caused involvement of regional lymph node and was classified as level-III, T4 and level-IV, T4 (1). In case of no treatment, B16F10 melanoma evolved to its metastatic stage and killed mice within weeks after inoculation, as reported previously (20).

**Figure 1:**
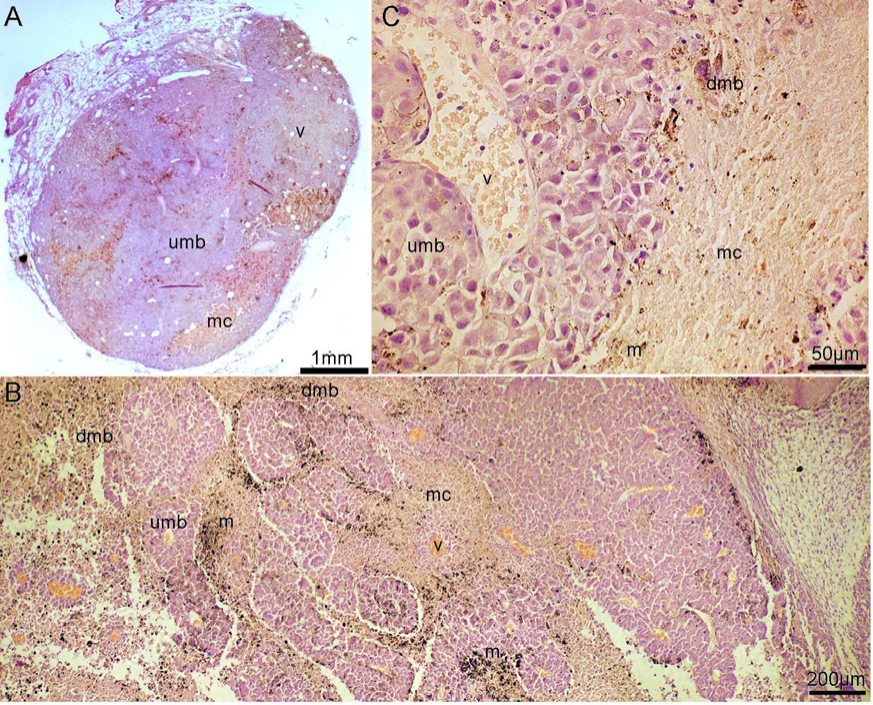
Melanoma growth was accompanied by progression of undifferentiated melanoblasts (umb) surrounding neoangiogenic vessels (v). Subsequently umb differentiated to melanoblasts (dmb) that produce melanin (m) as well as to melanocytes (mc) that form tumor stroma. (**A**) an entire (intact) tumor 8 days after inoculation, a prevalence of undifferentiated cells can be clearly seen. (**B,C**) a differentiated melanin-rich tissue was formed (micrographs on different magnifications were taken 15 days after cell inoculation).

Melanoma tumor at the beginning of its growth was characterized as a solid tumor with high angiogenesis and huge accumulation of undifferentiated melanoblasts (umb) around newly formed vessels, which does not produce much melanin (granules of melanin were rare on histological sections but the tumor itself was of black color) (Figure 1A). Further growth of the tumor was characterized by prominent angiogenesis with vessels surrounded by undifferentiated melanoblasts, and differentiation of melanoblasts to both melanocytes and their contribution to formation of tumor stroma (judged by melanin granules) cells was noticed (Figure 1). A subsequent growth caused the occurrence of internal necrosis areas surrounded with prominent lymphocyte infiltrates in the main tumor and formation of metastasis, which caused death (not shown). Based on the observed data, we subdivided melanoma tissue into a few regions according to Serov et al (21): undifferentiated melanoblasts, usually forming islets round vessels; differentiated melanoblasts producing melanin; and regions of tumor stroma (connective tissue).

### Glycomarkers of different melanoma regions

**Figure 2:**
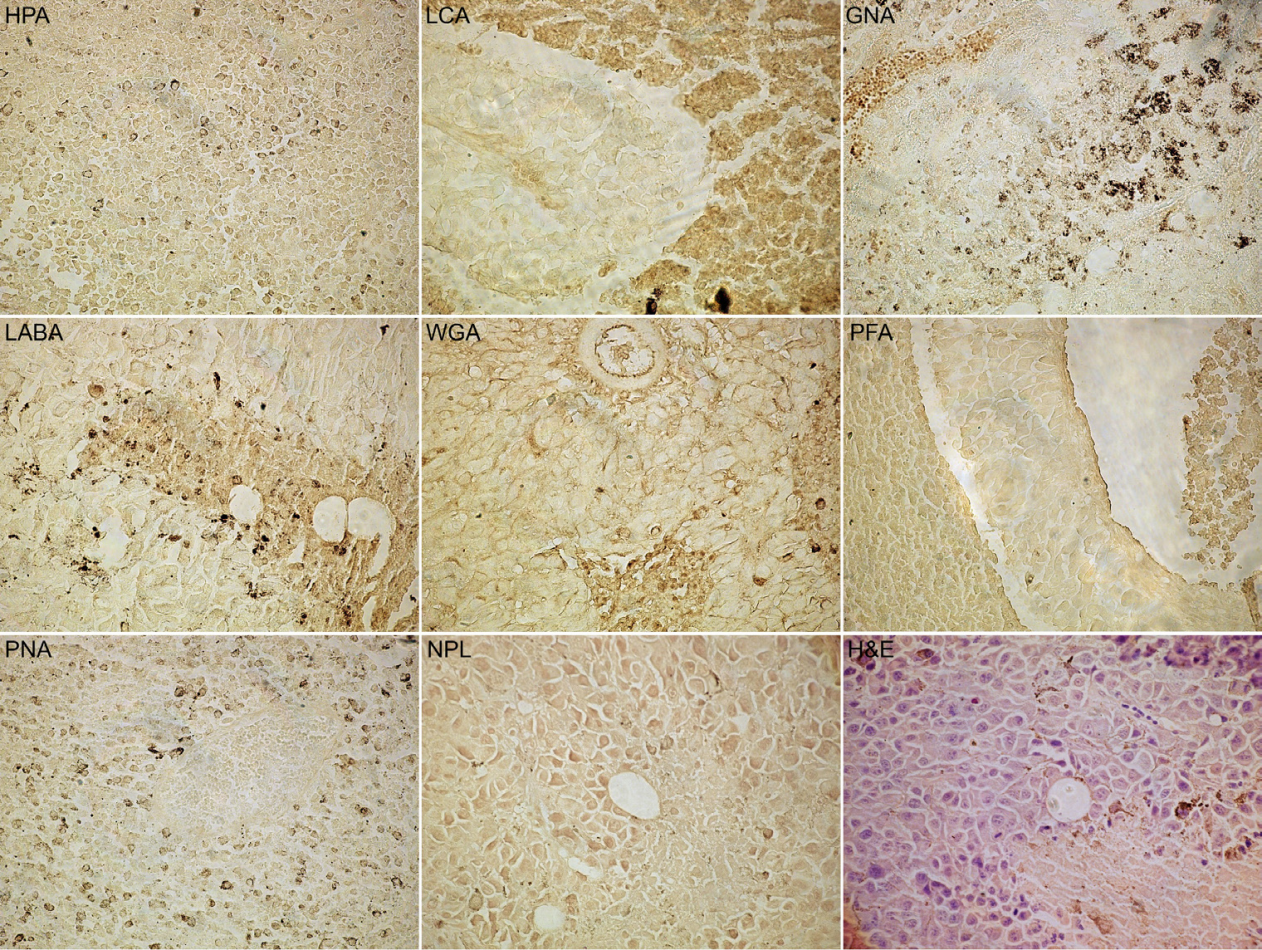
Lectin-histochemical analysis of different tumor regions. Lectins used for staining are indicated at top left. Lectin signal is in brown color. Hematoxylin and eosin staining is shown for the same tumor region as for NPL (subsequent slices were used). Objective ×10.

A set of lectins, binding specific tumor-related or cell death-related glycans were used to screen for an ability to bind distinct melanoma regions. Our goal was to find the lectin that would selectively bind to big multinucleated (with multiple nuclei) metabolically active undifferentiated melanoblasts by forming surrounding coating around neoangiogenic vessel, since they initiate the process of melanoma progression and possess the primarily danger. The lectins LABA (16,22) and PFA (23) used in the current work were discovered and GNA and NPL were characterized in our laboratory and were shown to possess an affinity toward altered, tumor-related glycoepitops, while NPL lectin was shown to be a promising marker of dying cells (24). The screened lectin panel showed preferential binding with distinct parts of melanoma tumors, allowing discrimination of tumor part based on their glycan composition (Figure 2, Table 1). PFA lectin demonstrated high specificity toward endothelium of neoangiogenic vessels. PNA lectin (specific to desialylated glycoepitops, namely to TF-antigen, is widely used in cancer research and diagnostic histopathology) (25) showed preferential binding with differentiated melanoblast and melanocytes in tumor stroma. GNA and especially NPL lectins (both specific to oligomannose-rich glycans) showed preferential binding with regions of undifferentiated melanoblasts, which was especially noteworthy for NPL lectin. Thus, we selected NPL for further experiments of fluorescent visualization of malignant melanoma tissue.

### Creation of lectin conjugates with fluorescent rare-earth nanoparticles

**Figure 3:**
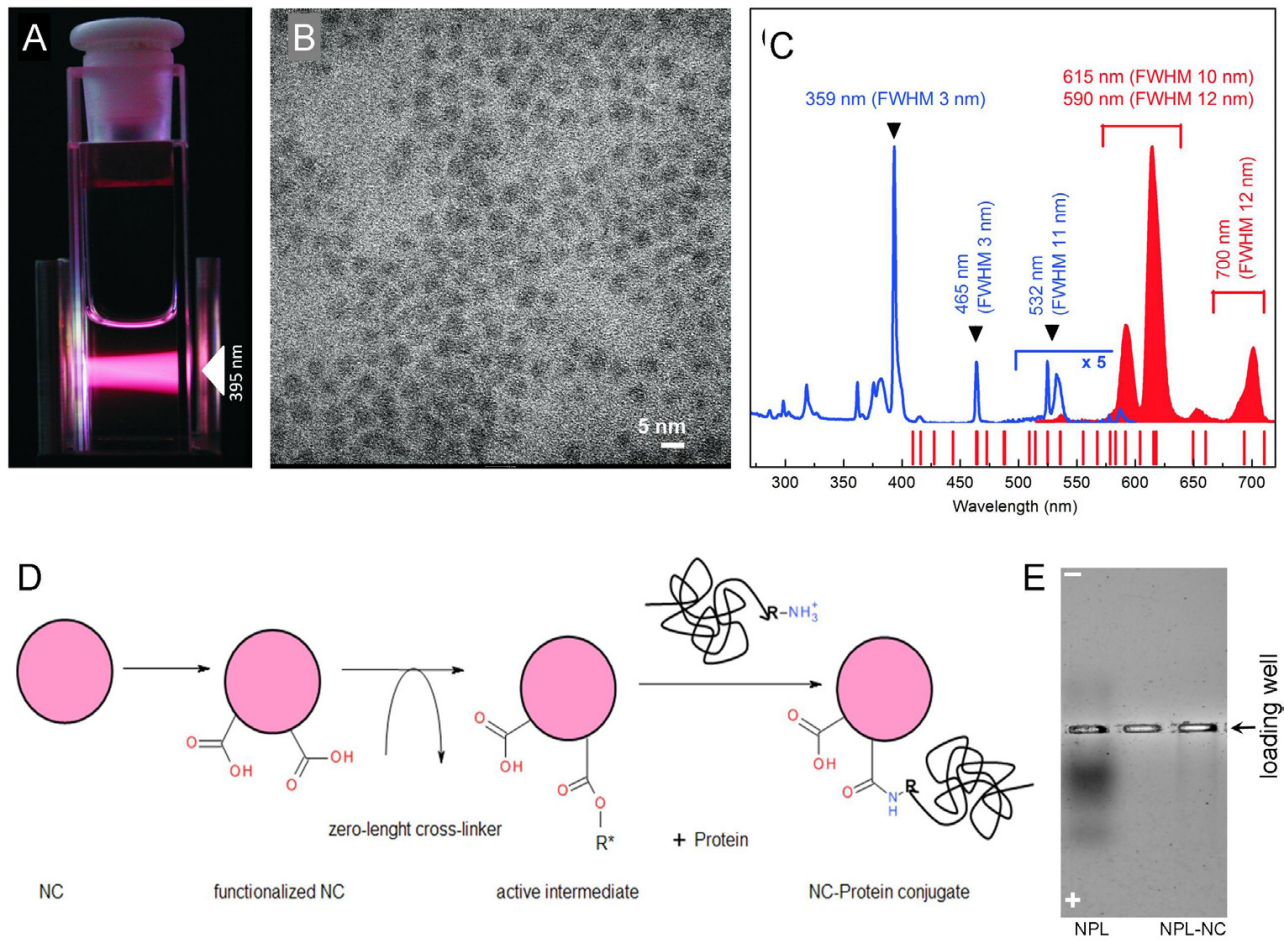
Properties of NPL- NaGdF_4_:Eu^3+^ nanocrystal (NC). (**A**) NC fluorescence is induced by 395 nm wavelength; (**B**) HRTEM image of NaGdF_4_:Eu^3+^ crystals revealed the diameter of 5 nm; (**C**) excitation and emission spectra of NaGdF_4_:Eu^3+^ NC; (**D**) scheme of NC conjugation with protein via zero-length cross linking reaction. (**E**) agarose gel electrophoresis of NPL protein and its conjugates with NC after extensive dialysis. No unbound protein was detected.

Fluorescent NaGdF_4_:Eu^3+^ NC were chosen for this study according to their excitation levels with conventionally available excitation light source and their ability to produce red and near-IR fluorescence, which we detected with the use of standard CCD cameras or other equipment. This matrix can be easily modified to emit light in NIR-IR range by simple exchange of lanthanide ions (ie, to Er or Tm) and can be potentially used for visualization of deep layers of tissues in vivo. NaGdF_4_:Eu^3+^ NC had hexagonal structure with mean diameter of 5 nm, as well as high photostability, and provided high fluorescence intensity in the red region (590 nm with full width at half maximum [FWHM] 12 nm and 615 nm with FWHM of 10 nm) and NIR region (700 nm with FWHM of 12 nm) (Figure 3, A and B). The surface of NC was modified according to our patent-pending method to make NCs water dispersible and to contain free carboxylic groups, which were further coupled to the targeted protein (NPL) via zero-length crosslinking reaction, using 1-ethyl-3-(3-dimethylaminopropyl)carbodiimide hydrochloride (Figure 3D). Unbound chemicals and uncoupled proteins were purified by dialysis and control of unbound proteins was done using 1% agarose gel electrophoresis. Free NPL protein was freely migrating in the agarose gel under the electric field, while NPL-NaGdF_4_:Eu^3+^ NC conjugates remained at their loading place and no traces of unbound proteins were detected (Figure 3E). NPL- NaGdF_4_:Eu^3+^ NC conjugates, which we created, were used for imaging of paraffin sections of melanoma tumors.

### Imaging of melanoma tissue using fluorescent NPL-NaGdF_4_:Eu^3+^ nanocrystals

**Figure 4:**
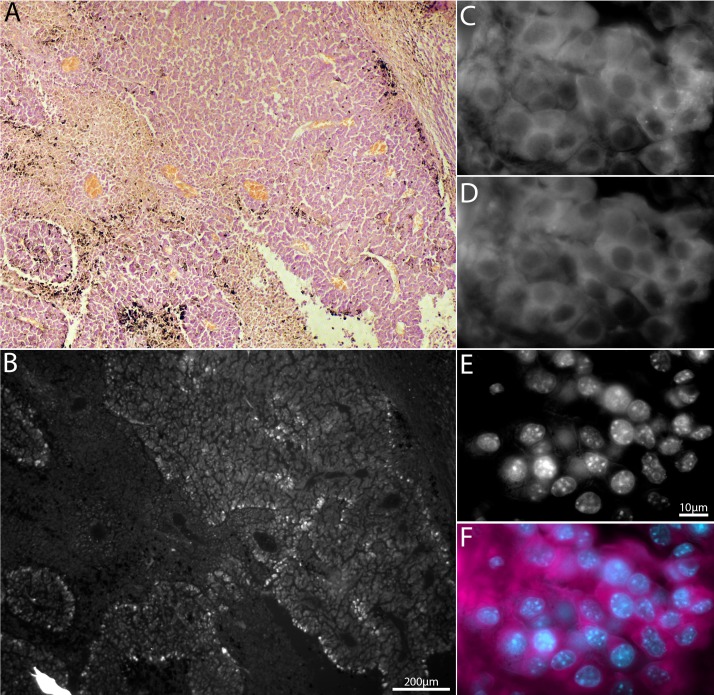
Visualization of undifferentiating neovascularized melanoma regions with NPL-NaGdF4:Eu^3+^ nanocrystals (NC). Melanoma slice stained with hematoxylin and eosin (**A**) is compared with sequential slice stained with NC conjugates, using near-infrared (NIR) fluorescence detection mode (**B**). NC provided two fluorescence peaks and were imaged using ex. 546/12nm, em. 610/60nm (a typical red fluorescent signal) with exposure of 500 ms (**C**); the same samples emitting at 690/50 nm (a NIR fluorescence, imaging using Carl Zeiss filter set 50) with exposure of 1100 ms were imaged at (**D**); cell nuclei of the sample in (**C**) and (**D**) were counterstained with 4',6-diamidino-2-phenylindole dihydrochloride (**E**); (**F**) merged images D and E.

Both water- and organic solvent-based mounting media were tested while mounting histological slides stained with conjugated NaGdF_4_:Eu^3+^ NC, and Canada-balsam based media showed excellent results for preserving the slides; NaGdF_4_:Eu^3+^ NC were compatible with xylene-containing mounting systems. NPL-NaGdF_4_:Eu^3+^ provided excellent signal-to-noise ratio when staining melanoma tumor and discriminating regions of undifferentiated melanoblasts and neoangiogenesis (Figure 4B) as compared to conventional HE staining (Figure 4A). Even after dozens of minutes of intense examination, the signal was not fading out. A repeated test for intensity showed that even after 1 and 6 months fluorescent signal did not exhibit any degradation. NPL-NaGdF_4_:Eu^3+^ NC provided strong signal in both red (Figure 4C) and NIR region (Figure 4D) and could be easily combined with DAPI counterstaining (Figure 4 E,F) for cell nuclei visualization.

## Discussion

This is the first study demonstrating the effectiveness of using rare-earth based NC for visualization of melanoma tumors in the near-IR spectral range. Selection of appropriate NC with strong emission in the IR is expected to allow future effective tracing of superficial tumors like melanoma with the aim to localize all tumor tissues and effectively remove it during surgery, while not destroying normal tissue. Small size of NC, their low toxicity (our currently unpublished data) and availability of specific lectin-based glycan markers providing reversible binding pattern should allow possible injections of NC-based labels and their subsequent removal from the organism.

It should be mentioned that both NPL and GNA lectins, which selectively label undifferentiated melanoblasts, are both specific to oligomannose glycans, namely oligomanose 5-9 glycans found as immature components of N-glycan biosynthesis pathways in the endoplasmic reticulum (ER) (26). Both have been previously reported as promising markers of apoptotic cells (24,27) and it has been shown that ER membranes contribute to compensation of cell surface membrane loss at apoptotic blebbing (24). Since the observed melanoblasts were typical undifferentiated cells with high metabolism (note multiple nucleoli seen in Figure 4 E,F), we can assume that they expose immature ER-related glycans on their surface due to incomplete N-glycan biosynthesis. A new group of mannoside-based compounds that blocks interactions involving oligomannose glycans was described recently (7,28). These compounds are in high demand as they can block the binding of *E coli* oligomanno-specific adhesine with host cell surface and are foreseen to be novel types of drugs for preventing bacterial re-infection cycles and host cell colonization during uropahtogenic infections and Crohn disease, both contributed to by *E coli* host cell invasion (29,30). The availability of compounds that would reversibly detach the NPL-NaGdF_4_:Eu^3+^ NC from their bound target is especially important for effective removal of NC from the organism after diagnostic procedure.

It is worth mentioning that although NIR irradiation of 710 nm produced by the studied NC cannot be detected by naked eye, it can be easily detected with conventional CCD and CMOS cameras (as well as FL4 detectors in most flow cytometers), whereas NC fluorescence in the red region (590 and 610 nm) can be easily observed by human eye under fluorescent microscope. Thus, the current finding can allow the creation of NIR NC targeted to label specific tumor regions based on altered glycoprofile. Imaging of such crystals after intravenous injection will allow a precise determination of tumor margins before its removal, while available synthetic glycan compound should allow making the binding reversible and removing NC from the organism after imaging. In conclusion, we showed that specific lectins can be effectively used to discriminate regions of melanoma and that their conjugates with rare-earth fluorescent nanocrystals provided efficient visualization of malignant melanoma tumors.
